# Associations of physical activity and screen time with adolescent idiopathic scoliosis

**DOI:** 10.1265/ehpm.23-00004

**Published:** 2023-09-28

**Authors:** Liwan Zhu, Shouhang Ru, Wanxin Wang, Qiufen Dou, Yanzhi Li, Lan Guo, Xiaosheng Chen, Weijun Wang, Wenyan Li, Zhixiang Zhu, Lei Yang, Ciyong Lu, Bin Yan

**Affiliations:** 1 Department of Medical Statistics and Epidemiology, School of Public Health, Sun Yat-sen University, Guangzhou, China; 2 Department of Spine Surgery, the First Affiliated Hospital of Shenzhen University, Shenzhen, China; 3 Department of Spine Surgery, the Shenzhen Second People’s Hospital, Shenzhen, China; 4 Shenzhen Youth Spine Health Center, Shenzhen, China

**Keywords:** Physical activity, Screen time, Adolescent idiopathic scoliosis, Case-control study

## Abstract

**Background:**

Adolescent idiopathic scoliosis (AIS) is the most common type of idiopathic scoliosis, affecting approximately 0.61%–6.15% adolescents worldwide. To date, the results on the relationship between moderate-to-vigorous physical activity (MVPA) and AIS were inconsistent, and the association between screen time (ST) and AIS remained unclear. This study aimed to describe MVPA and ST among adolescents, and to explore the independent and joint associations between PA, ST, and AIS.

**Methods:**

A frequency-matched case-control study based on the 2021 Chinese School-based Scoliosis Screening Program in Shenzhen city, south China, was conducted. The research involved 494 AIS patients (aged 9–17 years) and 994 sex- and age-matched healthy controls. MVPA and ST were measured using a self-administered questionnaire. Logistic regression models estimated associations between PA, ST, and AIS.

**Results:**

Compared to subjects meeting the recommended 60-min daily of MVPA, adolescents reporting daily MVPA time less than 60 min had 1.76 times higher odds of experiencing AIS (95% CI: 1.32–2.35) and adolescents reporting daily MVPA in inactive status had 2.14 times higher odds of experiencing AIS (95% CI: 1.51–3.03). Moreover, participants reporting ST for 2 hours or more had 3.40 times higher odds of AIS compared with those reporting ST less than 2 hours (95% CI: 2.35–4.93). When compared with the adolescents reporting both ST and MVPA meeting the guidelines recommended times (ST < 2 h and MVPA ≥ 60 min/day), those reporting both ST ≥ 2 h and MVPA in inactive status are 8.84 times more likely to develop AIS (95% CI: 3.99–19.61).

**Conclusions:**

This study reported that the insufficient MVPA, especially MVPA in inactive status, and excessive ST were risk factors for AIS. Additionally, the joint effects of insufficient MVPA and excessive ST probably increase the risk of AIS.

**Supplementary information:**

The online version contains supplementary material available at https://doi.org/10.1265/ehpm.23-00004.

## Background

Idiopathic scoliosis (IS) is a three-dimensional spinal deformity with lateral spine curvature with unknown cause [1], accounting for more than 80% of all scoliosis cases [2]. Adolescent idiopathic scoliosis (AIS) is the most common type of IS, mainly involving adolescents between 10 and 18 years of age. Epidemiological studies reported that IS affected approximately 0.61%–6.15% adolescents worldwide [3–9], and the ratios of IS in girls to boys increased from 2.7 to 8.4 following the aggravated spinal curvature [7]. Without proper intervention, severe IS may lead to cardiorespiratory impairment and related-morbidity due to lung restriction, and the severity of impairment has been shown to correlate with the degree of the spinal deformity [10–12]. At the same time, the progress of IS can cause significant deformities and psychosocial disturbances [13, 14], further leads to significant mental disorders including moderate depression, insomnia, and even somatization [14, 15]. Etiological researches suggested that IS may be a multifactorial disease [16, 17], yet the exact etiopathogenesis of IS is still unclear [18]. Of note and interest, research have pointed that environmental factors, such as physical activity, are involved in the etiopathogenesis of AIS [16].

Physical activity (PA) is well-known to provide physical beneficial effect for children and adolescents, including aerobic fitness, muscle strength, motor development, reducing cardiometabolic risk and preventing other chronic disease [19–21]. The latest studies reported that adequate PA is the key factor that may positively influences skeletal health in adolescents [22, 23]. Moreover, both high-intensity and low-intensity activity are associated with bone health [24], and the volume of PA may be more important than intensity [25]. There are robust evidence supported that greater amounts and higher intensities of physical activity are associated with improved health outcomes [26]. The World Health Organization (WHO) 2020 *Guidelines on Physical Activity and Sedentary Behavior* provided latest recommendations for children and adolescents (aged 5–17 years) that an average of 60-min of moderate-vigorous physical activity (MVPA) daily is many of benefits for physical and mental health [27]. The MVPA refers to PA performed at >3 metabolic equivalents (METS) [28]. A longitudinal study based on the IDEFICS/I.Family study reported that meeting the MVPA recommendation (daily MVPA meeting 1 h per day) kept the beneficial effect on bone strength during the observational period [29]. However, based on the current literature, it remains inconsistent conclusions whether MVPA is related to AIS and, if so, whether adolescents with AIS are less or equally active than their healthy peers [30–36]. At the same time, evidence indicated that many of the health-related benefits are observed among people with daily MVPA beyond 60 min [26]. Previous studies assessing PA have mainly focused on the levels of PA (e.g., high-intensity PA, moderate-intensity PA and low-intensity PA), and few studies have assessed the adequacy of daily PA and its association with AIS [30, 33–36]. Thereby, more studies are needed to further explore the relationship between PA and AIS.

Over time, digital media have evolved about a major part of life for current young generation. Several studies explore the physical (e.g., poor sleep, obesity) [37–43], psychological (e.g., depressive symptoms and suicidal behavior) [44–46], social and neurological adverse health consequence (e.g., less social support [47], low life satisfaction [48]) of excessive screen time (ST). At the same time, ST, mainly small-screen handheld devices, may result in affecting adolescents’ orthopedics, including head inclination and low bone mineral density (BMD) [49, 50]. However, the association of ST and AIS is poorly understood.

To date, the results on the relationship between PA and AIS were inconsistent. Additionally, little researches have examined the association between ST and AIS among Chinese. Furthermore, studies have reported that the joint effects of PA and ST can further aggravate the risk of certain health problems (e.g., overweight, sleep quantity and mental health) as compared to their independent effects [51–54]. Therefore, the aim of this study was to use case-control design to describe MVPA status among adolescents by evaluating whether it fulfill the 2020 WHO recommendations. Meanwhile, we explored the potential association between PA, ST and AIS, and joint effects of PA and ST.

## Methods

### Study design and participants

This case-control study was implanted based on the 2021 Chinese School-based Scoliosis Screening Program (CSSSP) conducted in Shenzhen Youth Spine Health Center (SYSHC) of the Shenzhen Second People’s Hospital (Shenzhen, China), a public service corporation that has established a scoliosis screening system for adolescents’ spine health records and a special clinic for further evaluation, diagnose and treatment for the students who screened positive (suspected of scoliosis). The CSSSP was an school scoliosis screening program for IS using a national scoliosis screening standardized protocol (GB/T 16133-2014) [55], collecting population-based scoliosis-related data every year since 2013. Students in primary schools (i.e., grades 4–6), junior high schools (i.e., grades 7–9), and senior high schools (i.e., grades 10–12) were invited to participate in the screening program voluntarily. School screening examinations included visual inspection and the Adams forward bending test (FBT) combined with scoliometer measurement. Children and adolescents who were positive in the screening (suspected of scoliosis) were referred to hospital for further diagnosis. Each student participating in the screening was assessed by two independent therapists separately. More detailed methods and processes for scoliosis screening could be found in our previous published study [56].

All patients who visited this hospital were invited in the current study. Data were collected from April 2021 to May 2022. The diagnosis of AIS was confirmed by both clinical assessment and standard whole standing postero-anterior full-spine radiographs. Scoliotic curve magnitude was evaluated by measuring Cobb’s angle on the standard whole full-spine film. The inclusion criteria for AIS group include: 1) aged 9 to 17 years, 2) the Cobb’s angle of the major curve ≥10° [57], and 3) willing to participate. The exclusion criteria include: 1) any other kinds of scoliosis (e.g., congenital scoliosis or neuromuscular scoliosis), 2) scoliosis of metabolic etiology, 3) congenital skeletal dysplasia, or 4) neuromuscular diseases. All measurements were performed by two independent experienced rehabilitation therapists and doctors. Control subjects were defined as children in the 2021 CSSSP who did not have any angle of trunk rotation (ATR) or significant physical signs. They were frequency-matched by age and sex with the AIS group. This study was conducted in accordance with the Declaration of Helsinki, and received ethical approval from the Shenzhen Second People’s Hospital Institutional Review Board (20211013002-fs01). All students were under 18 years of age, written informed consents were obtained from the parent or legal guardian of each participating student.

A total of 1,491 adolescents were recruited on a voluntary basis. After excluding students with invalid questionnaire (n = 3), the final analysis was carried out on a sample of 1,488 participants, including 494 AIS patients and 994 healthy controls, aged between 9 and 17.

### Physical activity

The physical activity was assessed using the International Physical Activity Questionnaire-Short Form (IPAQ-SF) [58], which asks about three types of activity (i.e., walking, moderate-intensity activities and vigorous-intensity activities) in the four domains (e.g., leisure time PA, work-related PA, etc.) during the past seven days [59]. Measurements of MVPA used four items from the IPAQ-SF that recorded the frequency (days) and duration (in minutes) of performing moderate (e.g., light-lifting, biking, double tennis) and vigorous (e.g., weight-lifting, aerobics) intensity activities. Then, the average daily MVPA was computed by following formula: ([moderate-intensity activities duration × days] + [vigorous-intensity activities duration × days])/7 [60]. Consistent with the WHO 2020 guidelines on PA and the 2018 PA guidelines for Americans (PAGA) [28, 61], the adolescents’ daily MVPA was classified into inactive status (daily MVPA < 10 mins), active not meeting the recommendations of MVPA (10 mins ≤ daily MVPA < 60 mins) and meeting the recommendations of MVPA (daily MVPA ≥ 60 mins) [62].

### Screen time

Participants were asked about the average number of hours spent on a weekday and weekend day for the following items: using computers, using smart-phone (or tablet), watching television, and electronic goods (e.g., handheld game consoles, console games, etc.). Participants responded to each question using 4-point scale: <30 min, 30–60 min, 60–90 min, or >90 min. The average daily ST was computed by taking a weighted average of the weekday and weekend ST ([weekday mean ST × 5] + [weekend mean ST × 2])/7 [63]. Regarding the Canadian 24-h movement guideline recommendation for adolescents, ST was then categorized as meeting (<2 h/day) or exceeding (≥2 h/day) [64].

### Covariates

Information about gender, age, ethnicity, family history of scoliosis, education level of father, education level of mother, myopia, academic pressure and dairy products intake were also collected. Family history of scoliosis was measured by asking subjects whether their families (e.g., brothers, parents, or grandparents) suffered from scoliosis. Smoking was assessed by a single item (“Have you used at least one cigarette during your lifetime?” Responses were coded as no = 1 and yes = 2). Academic pressure was assessed by asking the subject’s self-rating of his or her academic pressure (categorized into “above average = 1”, “average = 2”, and “below average = 3”). Dairy products intake was measured by asking subjects the question: “Have you eaten dairy products (e.g., milk, yogurt, cheese, etc.) in the past 1 year?” Anthropometric measurements included standing height (nearest 0.1 cm) and weight (accurate to 0.1 kg) were subjects self-reported. Body mass index (BMI) using the standard formula (kilograms per meter squared) was then calculated.

### Statistical analysis

Continuous variables were presented as means (standard deviation, SD) and categorical variables were presented as number (percentages, %). Additionally, based on the categorizations of MVPA and ST, the joint variable of MVPA and ST was categorized into six categories [51]: (1) recommended ST and MVPA (ST < 2 h/day and MVPA ≥ 60 mins/day), (2) recommended ST but MVPA active not meeting the recommendations of MVPA (ST < 2 h/day and 10 mins/day ≤ MVPA < 60 mins/day), (3) recommended ST but MVPA inactive (ST < 2 h/day and MVPA < 10 mins/day), (4) excessive ST but recommended MVPA (ST ≥ 2 h and MVPA ≥ 60 mins/day), (5) excessive ST and MVPA active not meeting the recommendations of MVPA (ST ≥ 2 h and 10 mins/day ≤ MVPA < 60 mins/day) and (6) excessive ST and MVPA inactive (ST ≥ 2 h and MVPA < 10 mins/day).

The statistical analysis was performed using SPSS, version 26.0 and the figures were prepared using ggplot2 (version 3.3.6) package in R, version 4.2.1. First, descriptive analyses were conducted to describe the demographic characteristics among subjects, the chi-square (*x*^2^) test or independent *t*-test was used to compare the differences between case and control groups. Second, differences in ST, PA, and a combination of both among adolescents by case and control groups were determined using the chi-square (*x*^2^) test. Third, the univariable logistic regression (LR) models were used to preliminarily explore the association between PA, ST and AIS. Then, the multivariable LR models after adjusting for potential confounding factors were used to further explore the independent associations and joint effects of PA and ST on AIS among adolescents by the case and control groups, while odds ratios (OR), adjusted odds ratios (aOR), and 95% confidence intervals (CI) were calculated. All multivariable LR models were adjusted for the same confounding factors including family history of scoliosis, education level of father, education level of mother and BMI. In addition, we conducted a series of sensitivity analysis. First, we used the average daily MVPA and ST (continuous variables) instead of the status of daily MVPA and ST (categorical variables) in the models to evaluate whether the estimated association were similar to that of the main analysis. Second, we used the complete dataset (n = 1,488) in which the missing data were imputed with multiple imputation method. We created five imputed datasets, and the estimates from the association analysis were pooled. The statistical significance was set at *P* < 0.05 (two-sided).

## Results

### Demographic characteristics

As shown in Table 1, a total of 1,488 subjects were included in the study. Of these, 1,068 were girls (71.8%) and 420 were boys (28.2%). The mean age of the subjects was 13.6 (SD: 1.8) years.

**Table 1:** Characteristics of the study subjects (*N* = 1488).

**Variables**	**Total, *n* (%)**	**Case, *n* (%)**	**Control, *n* (%)**	P*
Total	1488 (100)	494 (100)	994 (100)	NA
Age (year, mean (SD))	13.6 (1.8)	13.6 (1.8)	13.6 (1.8)	0.941
Gender				
Boys	420 (28.2)	140 (28.3)	280 (28.2)	0.945
Girls	1068 (71.8)	354 (71.7)	714 (71.8)	
Body height (m, mean (SD))	1.62 (0.1)	1.62 (0.1)	1.61 (0.1)	0.106
Body weight (kg, mean (SD))	50.47 (11.5)	48.32 (9.3)	51.55 (12.3)	<0.001
BMI (kg/m^2^, mean (SD))	19.2 (3.3)	18.2 (2.7)	19.6 (3.5)	<0.001
Ethnicity				
Han	1406 (96.4)	475 (96.7)	931 (96.2)	0.587
Minorities	53 (3.6)	16 (3.3)	37 (3.8)	
Missing data	29	NA	NA	
Myopia				
No	425 (28.7)	128 (26.0)	297 (30.1)	0.098
Yes	1055 (71.3)	365 (74.0)	690 (69.9)	
Missing data	8	NA	NA	
Family history of scoliosis				
No	1355 (91.2)	431 (87.2)	924 (93.1)	<0.001
Yes	131 (8.8)	63 (12.8)	68 (6.9)	
Missing data	2	NA	NA	
Education level of father				
Secondary school	236 (16.0)	72 (14.6)	164 (16.7)	0.005
High school	318 (21.5)	103 (20.9)	215 (21.9)	
College/University	724 (49.0)	270 (54.7)	454 (46.2)	
Unknown	199 (13.4)	49 (9.9)	150 (15.3)	
Missing data	11	NA	NA	
Education level of mother				
Secondary school	284 (19.2)	92 (18.7)	192 (19.4)	<0.001
High school	370 (25.0)	105 (21.3)	71.6 (26.8)	
College/University	630 (42.5)	255 (51.7)	375 (38.0)	
Unknown	197 (13.3)	41 (8.3)	156 (15.8)	
Missing data	7	NA	NA	
Academic pressure				
Above average	37 (2.5)	14 (2.9)	23 (2.3)	0.288
Average	1137 (77.0)	364 (74.6)	772 (78.2)	
Below average	302 (20.5)	110 (22.5)	192 (19.5)	
Missing Data	12	NA	NA	
Dairy products intake				
No	116 (7.8)	51 (10.3)	65 (6.6)	0.010
Yes	1368 (92.2)	442 (89.7)	926 (93.4)	
Missing data	4	NA	NA	
MVPA time (min/week, mean (SD))	50 (77.1)	37.1 (48.7)	56.3 (86.9)	<0.001

NA, not applicable or no data available.

Abbreviations: BMI, body mass index.

There were 494 patients with AIS and 994 healthy adolescents in this study. The distribution of age and sex was balanced between two groups (*p* > 0.05). Compared with controls, cases had lower mean weight and BMI (*p* < 0.001). Moreover, 12.8% of cases claimed that someone of their families had scoliosis, which nearly twice times higher than that in controls (*p* < 0.001). There were no significant differences in the distribution of ethnicity, myopia, education level of father, academic pressure, or dairy products intake between case and control groups. Among 1,488 subjects, 18 (1.2%) were of missing information on MVPA variable, and 26 (1.7%) were of missing information on ST variable. These participants were excluded from the current analysis. Additionally, there was no significant difference in the distribution of population characteristics between the participants included and those who were not included in the analysis (*p* > 0.05).

### MVPA and ST

**Fig. 1:**
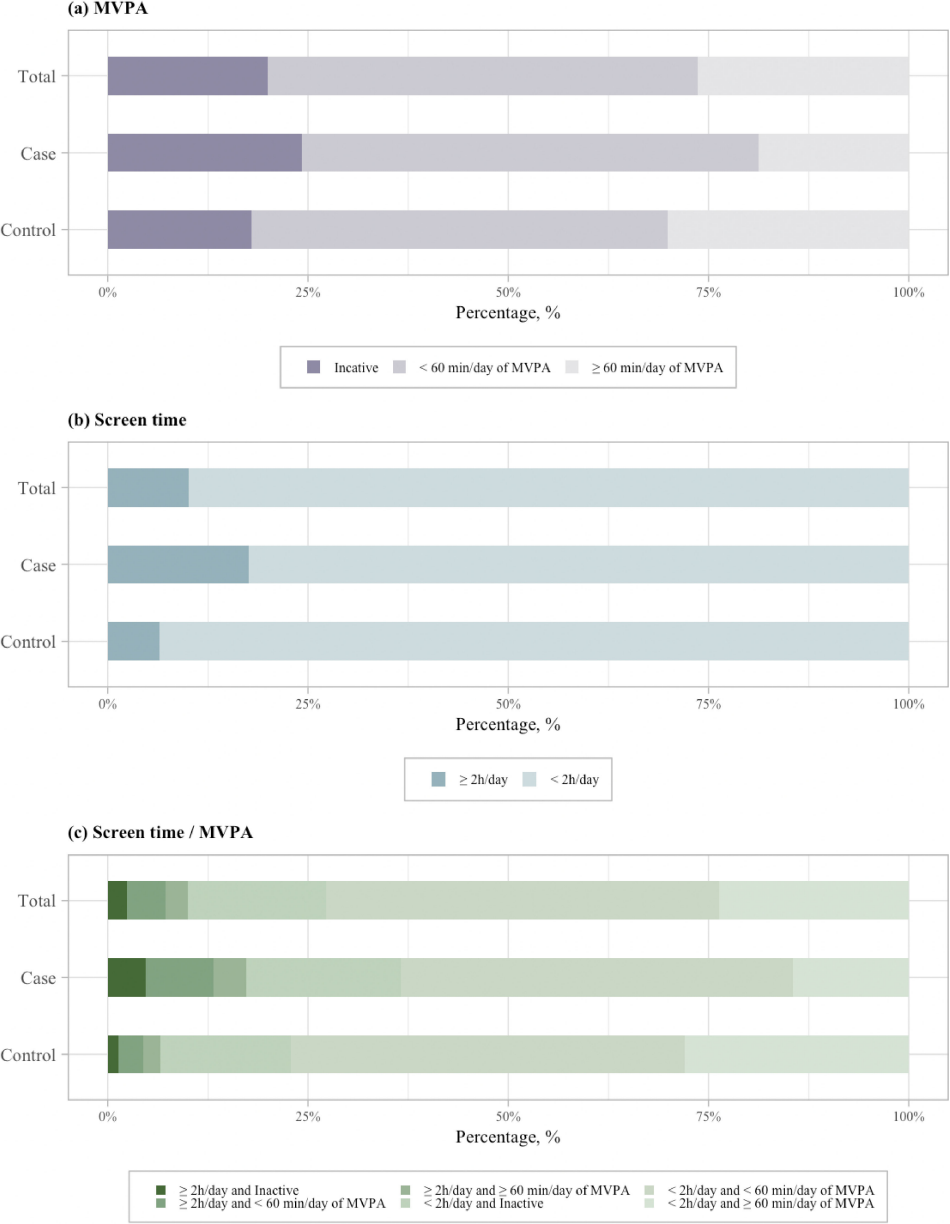
Comparison of MVPA, ST, and joint of ST and MVPA between case and control groups. Screen time/MVPA, a joint variable of screen time and MVPA; MVPA, moderate-vigorous physical activity.

Figure 1 presents the PA and ST status of participants by case and control groups. Overall, approximately 26.4% of the subjects met the recommended guidelines of 60 min of MVPA daily, while 20.0% of them reported daily MVPA in inactive status (Fig. 1a). In addition, nearly one-tenth (10.1%) of subjects reported 2 or more hours of daily ST time (Fig. 1b). Of the case group, only 18.8% of adolescents reported meeting the recommended 60-min MVPA per day, while 24.2% of subjects reported less than 10 min of MVPA every day. Compared to controls, cases had a lower proportion of meeting the MVPA recommended guidelines and higher proportion of MVPA in inactive status (*p* < 0.001). Notably, 17.6% of cases reported 2 or more hours of daily ST time, which nearly third times higher than that in controls (*p* < 0.001).

### Association of MVPA and ST with AIS

#### Independent associations

As shown in Table 2, binary LR was conducted to analyze independent associations between MVPA, ST and AIS. Regarding the univariable LR models, there were significant independent associations between MVPA, ST and AIS (*p* < 0.001). After adjusting for potential confounding factors, relative to subjects meeting the recommended 60-min MVPA, adolescents reporting daily MVPA time less than 60 min had 1.76 times higher odds of experiencing AIS (95% CI, 1.32–2.35) and adolescents reporting daily MVPA in inactive status had 2.14 times higher odds of experiencing AIS (95% CI, 1.51–3.03). Participants reporting ST for 2 or more hours had 3.40 times higher odds of AIS compared with those reporting ST less than 2 hours (95% CI, 2.35–4.93).

**Table 2:** Independent associations between screen time and physical activity and AIS.

**Variables**	**AIS**		

	**OR (95% CI)**	**aOR (95% CI)**	**Model 1, aOR (95% CI)**
Screen time			
<2 h/day	1.00 (reference)	1.00 (reference)	1.00 (reference)
≥2 h/day	3.08 (2.18–4.35)*	3.40 (2.35–4.93)*	3.40 (2.33–4.96)*
MVPA			
≥60 min/day of MVPA	1.00 (reference)	1.00 (reference)	1.00 (reference)
<60 min/day of MVPA	1.76 (1.33–2.32)*	1.76 (1.32–2.35)*	1.85 (1.37–2.51)*
Inactive	2.16 (1.55–3.01)*	2.14 (1.51–3.03)*	2.20 (1.53–3.15)*

OR: crude odds ratio.

aOR: model was adjusted for family history of scoliosis, education level of father, education level of mother and BMI.

Model 1 with AIS as dependent variables and both screen time and MVPA as independent variables, adjusted for family history of scoliosis, education level of father, education level of mother and BMI.

Abbreviations: OR, odds ratio; CI; confidence interval; aOR, adjusted odds ratio; MVPA, moderate-vigorous physical activity.

*P-value < 0.05.

#### Joint effect of MVPA and ST on AIS

According to combined PA and ST categories, the joint effect of PA and ST was categorized into six categories. Overall, only 23.7% of adolescents reported meeting both ST and MVPA recommendations, with nearly half (49.1%) reporting meeting ST recommendation with daily MVPA less than 60-mins, and 2.4% reporting not following the both ST and MVPA recommendations (Fig. 1c). Compared to controls, cases had a higher proportion of not following the both ST and MVPA recommendations (1.3% vs 4.8%, *p* < 0.001).

**Fig. 2:**
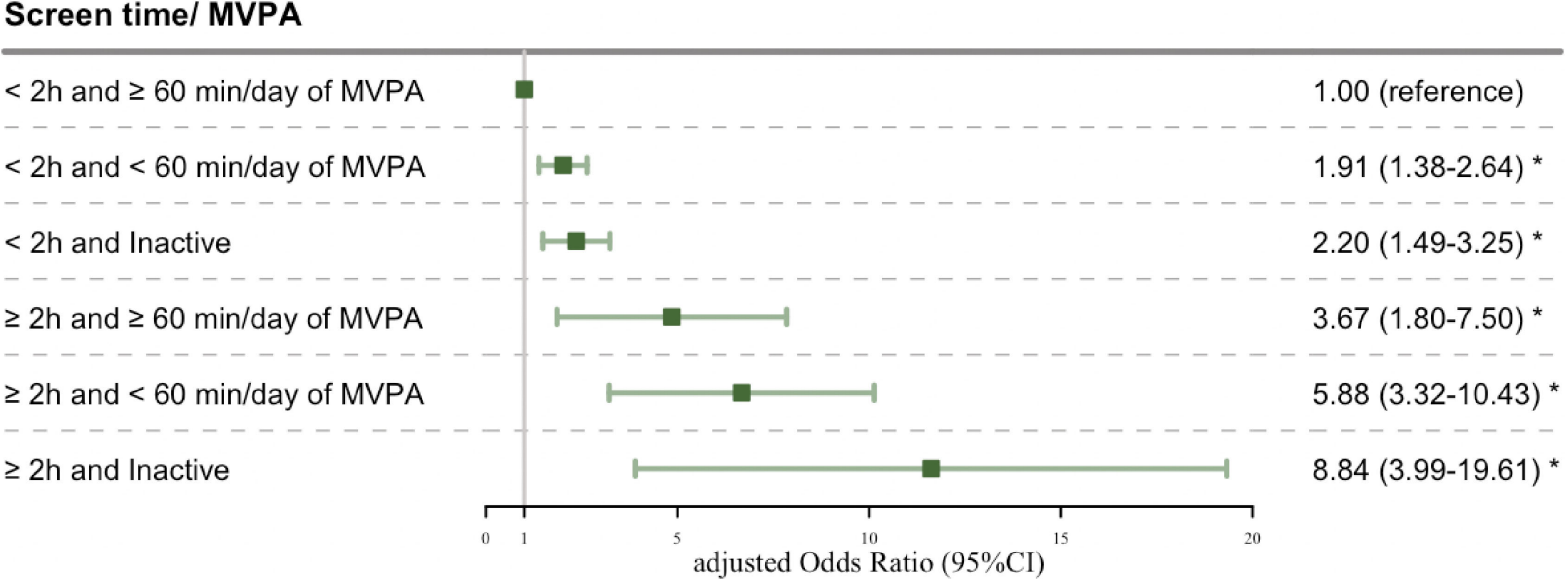
The joint associations of screen time and MVPA with AIS. Data expressed as adjusted odds ratio (aOR) and 95% confidence intervals. The model corresponds to the adjusted model, including family history of scoliosis, education level of father, education level of mother and BMI. Reference group: meeting the recommendation of daily ST and MVPA (aOR = 1). MVPA, moderate-vigorous physical activity; CI, confidence interval. *P-value < 0.05.

Figure 2 presents the aOR of adolescents being AIS using the multivariable LR model after adjusting for potential confounding factors, and the results showed that there was the obvious joint effects of PA and ST and AIS among adolescents (*p* < 0.05). When compared with the adolescents reporting both ST and MVPA meting the guidelines recommended times (ST < 2 h and ≥60 min/day of MVPA), the likelihood of experiencing AIS were increasing with increased ST and decreased MVPA time. Of note, adolescents who reported meeting the ST recommendation and MVPA in inactive status were 2.20 times likely to experience AIS (95% CI, 1.49–3.25), while those reporting exceed ST and MVPA in inactive status were 8.84 times (95% CI, 3.99–19.61).

The results about the association between MVPA, ST and AIS remained similar in the sensitivity analyses (supplementary Table 1).

## Discussion

We analyzed the association between MVPA and ST with AIS using frequency-matched case-control study among adolescents. Our results indicated that MVPA and ST were related to AIS. There was evidence that insufficient MVPA and excessive ST were both risk factors of AIS and they acted synergistically on the development of AIS.

In our study, we confirmed that MVPA was associated with AIS. Adolescents daily MVPA in inactive status or less than 60 mins were more likely to experience AIS, compared with those daily MVPA meeting the recommended 60-min.

Our results supported by a prospective population-based study, which indicated that children who performed more moderate/vigorous PA were 30% less likely to develop scoliosis [35]. A possible explanation for this association is that insufficient MVPA negatively affects bone health and increases the risk of scoliosis. The importance of intensity of PA on bone mineral density (BMD) and bone mineral content (BMC) has been well documented, particularly in children and adolescents [24, 65]. Meanwhile, many studies supported the link of abnormal BMD and low bone mineral mass to the etiopathogenesis in AIS patients [66–68]. Hence, improving bone quality and structure for the better bone strength during pubertal growth is essential for preventing AIS [30]. Adolescents with sufficient MVPA in high volume and intensity may have optimal bone health and less likely to have AIS. Furthermore, Tobias et al. showed that reduced muscle function may be a possible reason for this association between decreased PA and increased risk for developing scoliosis. The generalized muscle dysfunction may be one of the main potential abnormalities for scoliosis [35].

Nevertheless, there were studies that did not support our findings. In study conducted in 2020, it has shown that patients with AIS were as equally active as their peers without AIS [33]. This difference may be explained by the method of collecting and assessing PA information, and the selection of subject for the control group. There may be some selection bias from different study design.

To the best of our knowledge, few studies describing ST in AIS patients that includes a comparison with a representative healthy group. In this study, we examined the association between ST and AIS. The results revealed that excessive ST was related with AIS. That is, adolescents who reporting ST more than 2 hours were more likely to experience AIS. The intensive repetitive wrist and arm movement, and head inclination during using digital media will affect posture and cause musculoskeletal load result in discomfort symptoms [49], abnormal muscle function may increase the risk of AIS. Additionally, a systematic review including the literature of the past three decades provided strong support for a negative association between screen time and bone status in children and adolescents [69]. Screen time was negatively associated with bone density [50, 70], and generalized abnormal bone density have been reported in patients with AIS.

According to the outcomes of joint effects, we found that compared with adolescents reporting meeting the both ST and MVPA recommendations, those reporting different degreed of insufficient MVPA and excessive ST had a significantly higher odds of being AIS. It is critical to note the likelihood of developing AIS were increasing with increased ST and decreased MVPA time. Thus, increasing MVPA among adolescents with excessive ST might be a suitable approach toward reducing the risk of AIS. So far as we known, the current study is the first to explore the association between MVPA and ST combination and AIS. However, accumulating evidence indicates that the joint effects of excessive ST and insufficient MVPA more likely to lead to other health problems than their independent effects [52–54, 71–73]. A cross-sectional study using data collected in the large-scale Danish National Birth Cohort (DNBC) on spinal pain showed that the likelihood of moderate and severe spinal pain in pre-adolescents was progressively associated with increased ST and physically inactive, and the association of ST on spinal pain may be the strongest [71]. Moitra et al. revealed that the clustering of unhealthy lifestyle behaviors (i.e., low MVPA, excess ST and poor sleep) can increase pertinent risk of obesity [52]. Zhang et al. found the similar results [72]. Recently, a multi-ancestry meta-analysis of genome-wide association studies combined data for up to 703,901 individuals from 51 studies showed that 99 loci were associated with self-reported MVPA, ST and/or sedentary behavior at work. These loci yielded 104 independent association signals, implicating muscle and other organs [74]. This implies that future prospective longitudinal studies among children and adolescents need to further explore the causal association and potential mechanism between MVPA and ST, and AIS.

These findings may raise some enlightenment for enhancing spinal health among children and adolescents. Firstly, our findings provide essential information to better understand the associations between MVPA, ST, and AIS, which can be a significant basis for policymakers to improve appropriate program. Future public health strategies that aim to promote adolescents’ spinal health are highly recommended to target reducing ST and increasing MVPA simultaneously both in school and community (outside school) settings, as well as implementing policies at the national and local level. Furthermore, school health education on sufficient MVPA and advisable ST should be provided to help children and adolescents realize healthy lifestyles.

One of the strengths of this study is that it provides new insights into the association of screen time with AIS, which had rarely been examined. Furthermore, the current study explored the joint association of ST and MVPA with AIS controlling the potentially factors, which may present expedient direction for the guidance on appropriate preventing AIS.

The present study still has some limitations. A limitation is the case-control observational design of the study, and therefore the direction of causality cannot be directly inferred. The further prospective longitudinal studies aimed at describing the trajectory of MVPA, ST and AIS are needed to comprehensive grasp the associations and trends. Other limitations are the use of self-reported MVPA and ST, which is known to lead to a misestimate of their true statuses [75]. Nonetheless, the use of IPAQ for adolescents has rigorously evaluated for reliability and validity [76, 77].

## Conclusions

In conclusion, the study showed insufficient MVPA, especially MVPA in inactive status, and excessive ST are the risk factors for AIS. Concurrently, the combined effects of insufficient MVPA and excessive ST were significantly associated with AIS among adolescents. There is a need for a recommendation that children and adolescents are encouraged to be aware of the importance of both MVPA and ST in preventing AIS. Further prospective cohort study is needed to explore the mechanisms underlying these associations.

## Abbreviations

AIS: Adolescent idiopathic scoliosis
PA: physical activity
ST: screen time
MVPA: moderate-to-vigorous physical activity
WHO: The World Health Organization
METS: metabolic equivalents
BMD: bone mineral density
ATR: angle of trunk rotation
BMI: Body mass index
aOR: Adjusted odds ratios

## Supplementary information

**Additional file 1:** **Supplementary method:** The estimation of sample size. **Supplementary table 1.** Independent and combined associations between screen time and physical activity and AIS: sensitivity analyses.

## Declarations

### Availability of data and material

The data that support the findings of this study are available from the corresponding author upon reasonable request.
