# Supplementary material for: Associations of physical activity and screen time with adolescent idiopathic scoliosis
Source: Environ Health Prev Med. 2023 Sep 28;28:55. doi: 10.1265/ehpm.23-00004 (PMC10569969; doi:10.1265/ehpm.23-00004)
Supplement: Supplementary file 1 — Additional file 1: Supplementary method: The estimation of sample size. Supplementary table 1. Independent and combined associations between screen time and physical activity and AIS: sensitivity analyses. [file ehpm-28-055-s001.docx]

**Supplementary Material**

**Associations of physical activity and screen time with adolescent** **idiopathic scoliosis**

Liwan Zhu, Shouhang Ru, Wanxin Wang, Qiufen Dou, Yanzhi Li, Lan Guo, Xiaosheng Chen, Weijun Wang, Wenyan Li, Zhixiang Zhu, Lei Yang, Ciyong Lu, Bin Yan

**Supplementary method**

**The estimation of sample size**

The sample size was calculated as follows: $n=\frac{\left( Z_{\alpha}\sqrt{2\overline{pq}}+Z_{\beta}\sqrt{p_{0}q_{0}+p_{1}q_{1}} \right)^{2}}{\left( p_{1}-p_{0} \right)}$, *q_0_*=1-*p_0_*, *q_1_*=1-*p_1_*, *p*=(*p_1_*+*p_2_*)/2, and *q*=1-*p*. *p_0_* is the exposure rate of a related factor in the controls, and *p_1_* is the exposure rate of a related factor in the cases. We selected the variable “physical activity” to calculated the sample size^[1]^, and the needed number is 233 for the case group.

**Supplementary table 1.** Independent and combined associations between screen time and physical activity and AIS: sensitivity analyses

| Variables | AIS | |
| --- | --- | --- |
|  | OR (95% CI) | aOR (95% CI) |
| Screen time |  |  |
| < 2h | 1.000 (reference) | 1.000 (reference) |
| ≥ 2h | 3.024 (2.135-4.283) ^*^ | 3.288 (2.277-4.749) ^*^ |
| MVPA |  |  |
| ≥ 60 min/day of MVPA | 1.000 (reference) | 1.000 (reference) |
| < 60 min/day of MVPA | 1.742 (1.320-2.301) ^*^ | 1.706 (1.279-2.275) ^*^ |
| Inactive | 2.132 (1.510-3.012) ^*^ | 2.053 (1.432-2.942) ^*^ |
| Screen time/ MVPA^#^ |  |  |
| < 2h and ≥ 60 min/day of MVPA | 1.000 (reference) | 1.000 (reference) |
| < 2h and < 60 min/day of MVPA | 1.882 (1.385-2.558) ^*^ | 1.828 (1.331-2.509) ^*^ |
| < 2h and Inactive | 2.191 (1.512-3.174) ^*^ | 2.074 (1.411-3.047) ^*^ |
| ≥ 2h and ≥ 60 min/day of MVPA | 3.700 (1.904-7.187) ^*^ | 3.784 (1.870-7.658) ^*^ |
| ≥ 2h and < 60 min/day of MVPA | 5.197 (3.027-8.921) ^*^ | 5.441 (3.101-9.548) ^*^ |
| ≥ 2h and Inactive | 6.916 (3.239-14.766) ^*^ | 7.731 (3.476-17.198) ^*^ |
| MVPA time (min/week) | 0.994 (0.991-0.996) ^*^ | 0.994 (0.991-0.996) ^*^ |
| Screen time (min/week) | 1.201 (1.127-1.280) ^*^ | 1.235 (1.154-1.322) ^*^ |

OR: crude odds ratio.

aOR: model was adjusted for family history of scoliosis, education level of father, education level of mother and BMI.

Abbreviations: OR, odds ratio; CI; confidence interval; aOR, adjusted odds ratio; MVPA, moderate-vigorous physical activity.

^*^ *P*-value < 0.05.

^#^ Screen time/ MVPA: a combination of screen time and MVPA.

**References**

[1] Cui XL, Fang F, Chen YP. Investigation on life behavior of children aged 6-12 years with scoliosis in Zhengzhou. South China Journal of Preventive Medicine. 2022; 48: 337-9+43.
